# Molecular characterizations of *Giardia duodenalis* based on multilocus genotyping in sheep, goats, and beef cattle in Southwest Inner Mongolia, China[Fn FN1]

**DOI:** 10.1051/parasite/2022036

**Published:** 2022-07-06

**Authors:** Yin Fu, Heping Dong, Xiaokun Bian, Ziyang Qin, Han Han, Jiashu Lang, Junchen Zhang, Guanghui Zhao, Junqiang Li, Longxian Zhang

**Affiliations:** 1 College of Veterinary Medicine, Henan Agricultural University Zhengzhou 450046 China; 2 International Joint Research Laboratory for Zoonotic Diseases of Henan Zhengzhou 450046 China; 3 College of Veterinary Medicine, Northwest A&F University Yangling 712100 China; 4 Norman Bethune Health Science Center of Jilin University Changchun 130015 China

**Keywords:** *Giardia duodenalis*, Molecular characterization, Ruminants, Inner Mongolia

## Abstract

*Giardia duodenalis* is an important zoonotic parasite that causes economic losses to animal husbandry and threatens public health. In the present study, a total of 1466 fresh fecal samples were collected from sheep (*n* = 797), goats (*n* = 561) and beef cattle (*n* = 108) in Southwest Inner Mongolia, China. *Giardia duodenalis* was initially screened via nested polymerase chain reaction (PCR) targeting the β-giardin (*bg*) gene, and *bg*-positive samples were subjected to PCR amplification targeting the glutamate dehydrogenase (*gdh*) and triose phosphate isomerase (*tpi*) genes. A total of 4.0% of samples (58/1466) were positive for *G. duodenalis*, with a prevalence of 3.4% in sheep, 3.7% in goats and 5.2% in beef cattle. Three *G. duodenalis* assemblages (A, B, and E) were identified, with E as the prevalent assemblage. Four and one novel assemblage E sequences were obtained for the *gdh* and *tpi* loci, respectively and four assemblage E multilocus genotypes (MLG) were obtained. This study demonstrates high genetic variations in *G. duodenalis* assemblage E, and provides baseline data for preventing and controlling *G. duodenalis* infection in livestock in Inner Mongolia.

## Abstract (fr)

*Giardia duodenalis* est un parasite zoonotique important, qui cause des pertes économiques à l’élevage et menace la santé publique. Dans la présente étude, un total de 1466 échantillons fécaux frais ont été prélevés sur des moutons (*n* = 797), des chèvres (*n* = 561) et des bovins de boucherie (*n* = 108) dans le sud-ouest de la Mongolie intérieure, en Chine. *Giardia duodenalis* a été initialement criblé via une réaction en chaîne par polymérase imbriquée ciblant le gène de la β-giardine (*bg*), et les échantillons *bg*-positifs ont été soumis à une amplification par PCR ciblant les gènes de la glutamate déshydrogénase (*gdh*) et de la triose phosphate isomérase (*tpi*). Au total, 4,0 % (58/1466) des échantillons étaient positifs pour *G. duodenalis*, avec une prévalence de 3,4 % chez les ovins, 3,7 % chez les caprins et 5,2 % chez les bovins. Trois assemblages de *G. duodenalis* (A, B et E) ont été identifiés, E étant l’assemblage prédominant. Respectivement quatre et une nouvelle séquences de l’assemblage E ont été obtenues dans les loci *gdh* et *tpi*, et quatre génotypes multilocus (MLG) de l’assemblage E ont été mis en évidence. Cette étude montre des variations génétiques élevées dans l’assemblage E de *G. duodenalis* et fournit des données de base pour prévenir et contrôler l’infection à *G. duodenalis* chez le bétail en Mongolie intérieure.

## Introduction

*Giardia duodenalis* (synonym *G. intestinalis* and *G. lamblia*) is one of the most common intestinal pathogens in both humans and animals [25]. The symptoms of *Giardia*sis are diarrhea, abdominal pain and weight loss [1, 10, 30]. Livestock has been reported as a common reservoir of *G. duodenalis*, with an individual prevalence ranging from 0 to 73% [9, 17]. Although *G. duodenalis* infection is commonly asymptomatic, many reports of *Giardia*sis in calves, goats and lambs show decreased weight gain and impairment in feed efficiency, causing significant economic losses to the farm [1, 12, 29].

*Giardia duodenalis* has a complex assemblage with a classification that is based on sequence analyses. The genetic locus of small subunit rRNA (*SSU rRNA*) [2], beta-giardin (*bg*) [16], glutamate dehydrogenase (*gdh*) [4], and triose phosphate isomerase (*tpi*) is commonly used for PCR to characterize *G. duodenalis* [28]. Multilocus genotype (MLG) analysis based on *bg*, *gdh*, and *tpi* is widely used for identifying genetic variations in *G. duodenalis* [6, 8].

Thus far, eight assemblages (A–H) of *G. duodenalis* have been identified based on genetic analysis and specific hosts [19]. Assemblages A and B have low host specificity and can infect humans as well as several other vertebrates; there are three assemblage A subgroups (AI, AII and AIII) and subgroup AIII has only been found in wildlife. However, assemblages C–H seem to be host-adapted; of these, assemblages C and D are mainly found in canines, assemblage E in artiodactyls, assemblage F in felines, assemblage G in rodents, and assemblage H in seals and some aquatic mammals [5, 24]. Previous studies have shown that artiodactyls are predominately infected by assemblages A and E, and a few reports have described assemblage B in artiodactyls [32, 33].

*Giardia duodenalis* is widely distributed in sheep, goats, and cattle (including dairy cattle, beef cattle, and yaks) in China [17]. Inner Mongolia is the third largest province in China, and animal husbandry makes an important economic contribution to the area. In Inner Mongolia, there are only three reports of *G. duodenalis*, in sheep and Bactrian camels [6, 34, 37]. More investigations are needed to facilitate improved interventions and minimize the burden of *G. duodenalis* in livestock. The objectives of this study were to further investigate and expand the prevalence information on *G. duodenalis* in ruminants in Southwest Inner Mongolia, China.

## Materials and methods

### Ethical standards

Following the Chinese Laboratory Animal Administration Act of 1988, the research protocol was reviewed and approved by the Research Ethics Committee of Henan Agricultural University (Approval No. IRB-HENAU-20180914-01). Appropriate permission from farmers was obtained before collecting fecal samples, and no animals were harmed.

### Sample collection

**Figure 1:**
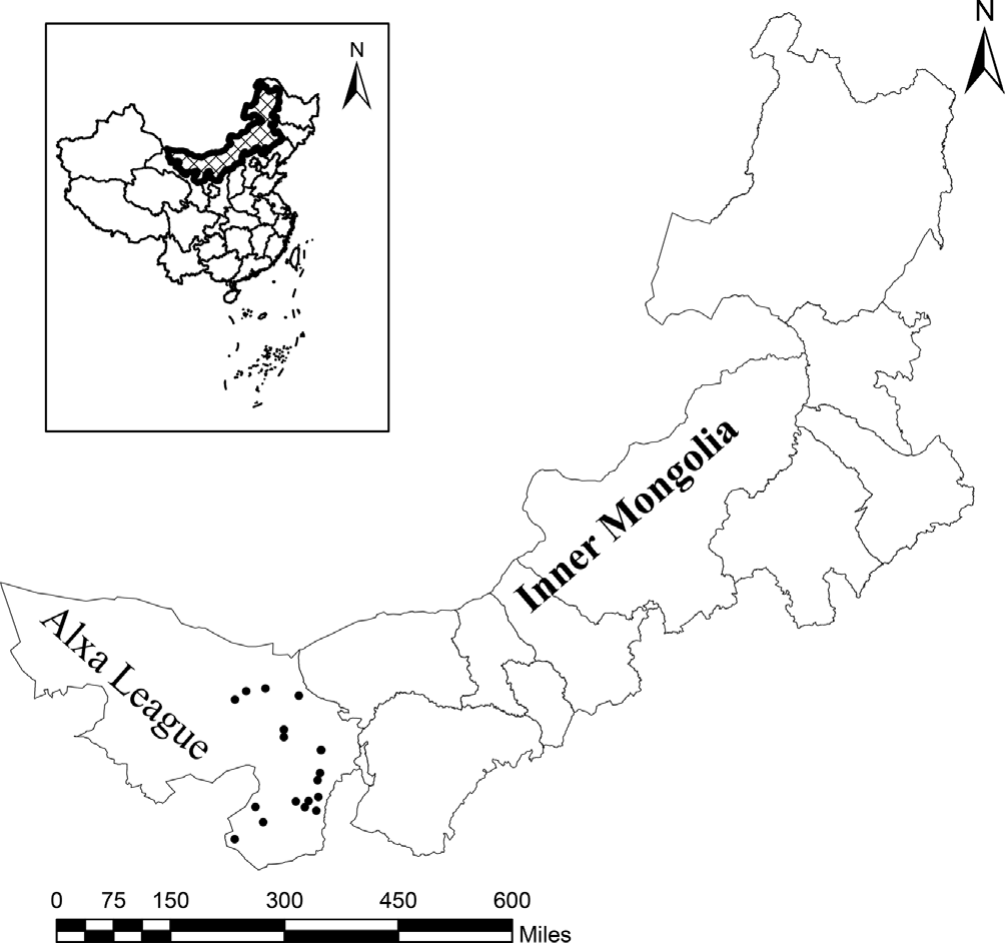
Location of the study area in Alxa League, Southwest Inner Mongolia, China. Sampling sites are marked by filled spots.

From October 2019 to July 2021, a total of 23 farms were chosen randomly in northwest Inner Mongolia, China (Fig. 1). A total of 1466 fresh fecal specimens were collected from sheep (*n* = 797), goats (*n* = 561), and beef cattle (*n* = 108), respectively (Table 1). Of these, 1083 were from more than 12-month-old livestock, and 383 were from 7–12 month-old livestock; 419 samples were collected in the summer, 289 in autumn and 758 in winter (Table 2). Fresh fecal samples were collected by rectal sampling from ruminants in pens, and samples were gathered from the top layer of feces when grazing livestock defecated on the ground to ensure that there was no contamination [27]. Samples were stored in clean bags and transported in foam containers under ice conditions. No abnormal fecal specimens were observed during sample collection.

**Table 1:** Sampling information and the occurrence of *G. duodenalis* in ruminants in Southwest Inner Mongolia, China.

Administrative region	Sampling site	Sample number	Positive % (no. positive/no. sampled)	Animal Species	Feeding models
Alxa Left Banner (Inner Mongolia)	1	20	10.0 (2/20)	Goat	Pastoral
	2	137	1.5 (2/137)	Sheep	Captive
	3	30	0	Goat	Pastoral
	4	45	0	Goat	Pastoral
	5	57	0	Goat	Pastoral
	6	21	0	Sheep	Pastoral
	7	51	5.9 (3/51)	Goat	Pastoral
	8	104	6.7 (7/104)	Sheep	Pastoral
	9	22	27.3 (6/22)	Sheep	Captive
		108	9.3 (10/108)	Beef cattle	Captive
	10	120	10.0 (12/120)	Goat	Pastoral
		9	0	Sheep	Pastoral
	11	158	0	Sheep	Pastoral
	12	35	11.4 (4/35)	Goat	Pastoral
		10	0	Sheep	Pastoral
	13	42	2.4 (1/42)	Goat	Pastoral
	14	29	6.9 (2/29)	Sheep	Pastoral
		15	0	Goat	Pastoral
	15	22	0	Goat	Pastoral
	16	69	2.9 (2/69)	Sheep	Pastoral
	17	15	0	Goat	Pastoral
	18	20	0	Goat	Pastoral
	19	118	0	Sheep	Captive
	20	20	0	Sheep	Captive
Alxa Right Banner (Inner Mongolia)	21	21	0	Sheep	Pastoral
	22	122	3.3 (4/122)	Sheep	Captive
	23	46	6.5 (3/46)	Sheep	Captive
Total		1466	4.0 (58/1466)		

**Table 2:** Prevalence of *G. duodenalis* under different conditions.

Factors	Category	Positive % (no. positive/no. sampled)		
		Sheep	Goats	Beef cattle
Feeding model	Pastoral	3.3 (11/332)	3.7 (21/561)	0
	Captive	3.4 (16/465)	0	9.3 (10/108)
Age group	6–12 months	2.3 (7/306)	1.6 (1/63)	35.7 (5/14)
	>12 months	4.1 (20/491)	4.0 (20/498)	5.3 (5/94)
Season	Summer	2.0 (7/348)	4.2 (3/71)	0
	Autumn	1.5 (2/137)	2.0 (3/152)	0
	Winter	5.8 (18/312)	4.4 (15/338)	9.3 (10/108)

**Table 3:** Intra-assemblage substitutions in *bg*, *gdh*, and *tpi* sequences within *G. duodenalis* assemblage E.

Sequence (no.)	Nucleotide positions									GenBank ID
***gdh***	51	72	105	166	210	215	282	327	455	
E9 (1)	C	C	C	A	G	T	T	T	G	KT698969
E45 (4)	–	–	–	–	–	–	–	–	A	KC960648
E46 (2)	–	–	–	–	–	–	G	–	–	MK442907
E47 (1)	–	–	T	–	–	–	–	–	G	KY655475
E48 (4)	–	–	–	–	–	–	G	–	G	MK442905
E49^a^ (1)	–	–	–	G	–	G	–	–	–	OL456202
E50^a^ (1)	–	–	–	–	–	–	–	G	–	OL456203
E51^a^ (2)	–	–	–	–	A	–	–	–	–	OL456204
E52^a^ (1)	T	T	–	–	–	–	–	–	G	OL456206
***bg***	68	110	173	275	401	416				
E1 (6)	C	C	A	C	G	C				MK610388
E27 (1)	T	T	G	–	–	T				MK610379
E35 (29)	–	–	–	–	–	T				MK610387
E36 (16)	T	–	–	–	A	T				MT108433
E37 (2)	T	–	–	–	–	T				MF671888
E38 (1)	T	–	G	–	–	T				MT713328
E39 (1)	T	–	–	–	–	–				MK610389
E40 (1)	T	–	–	T	–	T				LC484286
***tpi***	37	58	91	95	145	316				
E1 (1)	G	T	T	A	A	T				KY769102
E3 (2)	–	–	–	G	–	–				KY769100
E5 (1)	A	–	–	–	G	–				EF654686
E32^a^(1)	–	C	G	A	–	C				OL456207

N-dash (–) indicates that the sequence is the same as the reference sequence.

a Novel sequence.

**Table 4:** Multilocus characterization of *G. duodenalis* isolates based on the beta-giardin (*bg*), glutamate dehydrogenase (*gdh*), and triose phosphate isomerase (*tpi*) genes in hosts.

Serial number of samples	Host	Genotype or subtype			MLGs (*bg*-*gdh*-*tpi*)
		*bg*	*gdh*	*tpi*	
7	Goat	E35	E51	–	–
11	Goat	E35	E51	–	–
52	Sheep	E1	E45	–	–
60	Sheep	E39	E46	–	–
189	Goat	E1	E45	–	–
437	Goat	E35	–	–	–
446	Goat	E35	E48	–	–
447	Goat	E27	E48	E3	MLG-E1
466	Sheep	E35	–	–	–
484	Sheep	E35	–	–	–
485	Sheep	E35	–	–	–
488	Sheep	E35	–	–	–
489	Sheep	E35	–	–	–
491	Sheep	E35	–	–	–
506	Sheep	E35	–	–	–
561	Sheep	E35	–	–	–
562	Sheep	E35	–	–	–
563	Sheep	E35	–	–	–
564	Sheep	E35	–	–	–
573	Sheep	E35	E45	–	–
579	Sheep	E35	E49	E1	MLG-E2
580	Sheep	E35	–	–	–
597	Goat	E36	–	–	–
599	Goat	E36	–	–	–
603	Goat	E36	–	–	–
604	Goat	E36	–	–	–
605	Goat	E36	–	–	–
607	Goat	E36	–	–	–
612	Goat	E36	–	–	–
616	Goat	E36	–	–	–
620	Goat	E36	–	–	–
642	Goat	E35	–	E5	–
782	Goat	E36	–	–	–
791	Goat	E36	–	B	Mixed
793	Goat	E36	–	–	
800	Goat	E36	–	–	
836	Goat	E36	–	–	
880	Sheep	E35	E48	–	
894	Sheep	AI	–	–	
1061	Sheep	E36	–	–	
1062	Sheep	E36	–	–	
1109	Beef cattle	E38	–	–	
1137	Beef cattle	E37	E47	E3	MLG-E3
1139	Beef cattle	E35	E50	–	
1152	Beef cattle	E35	–	–	
1154	Beef cattle	E40	–	–	
1201	Beef cattle	E1	–	–	
1202	Beef cattle	E35	–	–	
1210	Beef cattle	E1	E9	–	
1212	Beef cattle	E35	E45	–	
1213	Beef cattle	E37	E46	–	
1375	Sheep	E35	E52	E32	MLG-E4
1430	Sheep	E1	–	–	
1439	Sheep	E35	–	–	
1441	Sheep	E1	–	–	
1503	Sheep	E35	–	–	
1532	Sheep	E35	E48	–	
1537	Sheep	E35	–	–	

N-dash (–) indicates that no data were obtained.

### DNA extraction and PCR amplification

The genomic DNA of each fecal sample was extracted using a commercial E.Z.N.A Stool DNA kit (Omega Bio-Tek Inc., Norcross, GA, USA), strictly following the specifications of the manufacturer. All the extracted DNA samples were stored at −20 °C.

*Giardia duodenalis* was initially screened via nested PCR amplification targeting the *bg* [7] gene, and then studied by a MLG analysis based on the *gdh* [4] and *tpi* [28] genes. After amplification, the DNA fragments were separated by agarose gel electrophoresis (1% agarose) stained with DNA Green (TIANDZ, Beijing, China) and observed using a Tanon 3500 Gel Image Analysis System (TANON, Shanghai, China). Amplified samples with the target band were selected as positive PCR production (*bg* is 511 bp, *gdh* is 520 bp, *tpi* is 530 bp).

### Sequence analysis

Positive PCR amplicons with the target band were sequenced by SinoGenoMax (Beijing, China). Bidirectional sequencing was chosen to ensure the veracity of sequences. The sequences in this study aligned with reference sequences from GenBank using ClustalX 2.1 (http://www.clustal.org/). Samples were amplified at the *bg*, *gdh* and *tpi* loci to form MLGs to further reveal genetic diversity. The same nomenclature system as in previous reports was used in naming *G. duodenalis* assemblage E subtypes at each genetic locus. Undesignated subtype sequences previously published and novel subtype sequences identified in this study were named accordingly as E36–E40 at the *bg* locus, E45–E52 at the *gdh* locus, and E32 at the *tpi* locus [6, 7, 22] (Table 3).

Phylogenetic analysis was conducted using the maximum composite likelihood model, and bootstrap values were calculated by analyzing 1000 replicates and the other chosen default parameters in MEGA 7.0 software (http://www.megasoftware.net/).

### Statistical analysis

A Chi-square test was performed, and 95% confidence intervals (CIs) were calculated using Crosstab in SPSS, version 24.0 (SPSS Inc., Chicago, IL, USA). A Pearson’s chi-squared test was used for comparisons between two groups, and *p* < 0.05 was considered statistically significant.

### Nucleotide sequence accession numbers

The representative nucleotide sequences were submitted to the GenBank at the National Center for Biotechnology Information under accession numbers: OL456202, OL456203, OL456204 and OL456206 for the *gdh* gene, and OL456207 for the *tpi* gene.

## Results

### Occurrence of *G. duodenalis* in ruminants

A total of 58 (4.0%) *G. duodenalis*-positive fecal samples were identified by the nested PCR analysis based on the *bg* gene, with 3.4% (27/797) in sheep, 3.7% (21/561) in goats and 9.2% (10/108) in beef cattle. The infection rates in winter were significantly higher than in summer (*p* = 0.009, 95% CI: 0.202–0.818) and autumn (*p* = 0.006, 95% CI: 0.115–0.747).

Among the positive samples in sheep, 11 were from pastoral sheep and 16 were from captive sheep, and there was no significant difference in *G. duodenalis* infection between pastoral and captive sheep (*p* = 0.922, 95% CI: 0.440–2.100). The *G. duodenalis* infection rate was significantly different between different age groups of beef cattle (*p* < 0.001, 95% CI: 2.399–40.770). There were no significant differences in prevalence of *G. duodenalis* among different age groups of sheep (*p* = 0.108, 95% CI: 0.211–1.183) and goats (*p* = 0.222, 95% CI: 0.041–2.292) (Table 2).

### Sequence and subtype analysis

A total of 58 *bg* sequences, 17 *gdh* sequences and 6 *tpi* sequences were obtained. Three kinds of assemblages were identified, including *G. duodenalis* assemblage A (*n* = 1), assemblage E (*n* = 56), and a mix of assemblages B and E (*n* = 1). Additionally, 4 samples were simultaneously amplified at all three intra-assemblage variation genetic loci (*bg*, *gdh*, *tpi*), forming 4 novel assemblage E MLGs (MLG-E1 to MLG-E4). The MLG-E2 and MLG-E4 sequences were obtained from sheep; the MLG-E1 sequences were obtained from goats, and the MLG-E3 sequences were obtained from beef cattle (Table 4).

### Phylogenetic analysis

**Figure 2:**
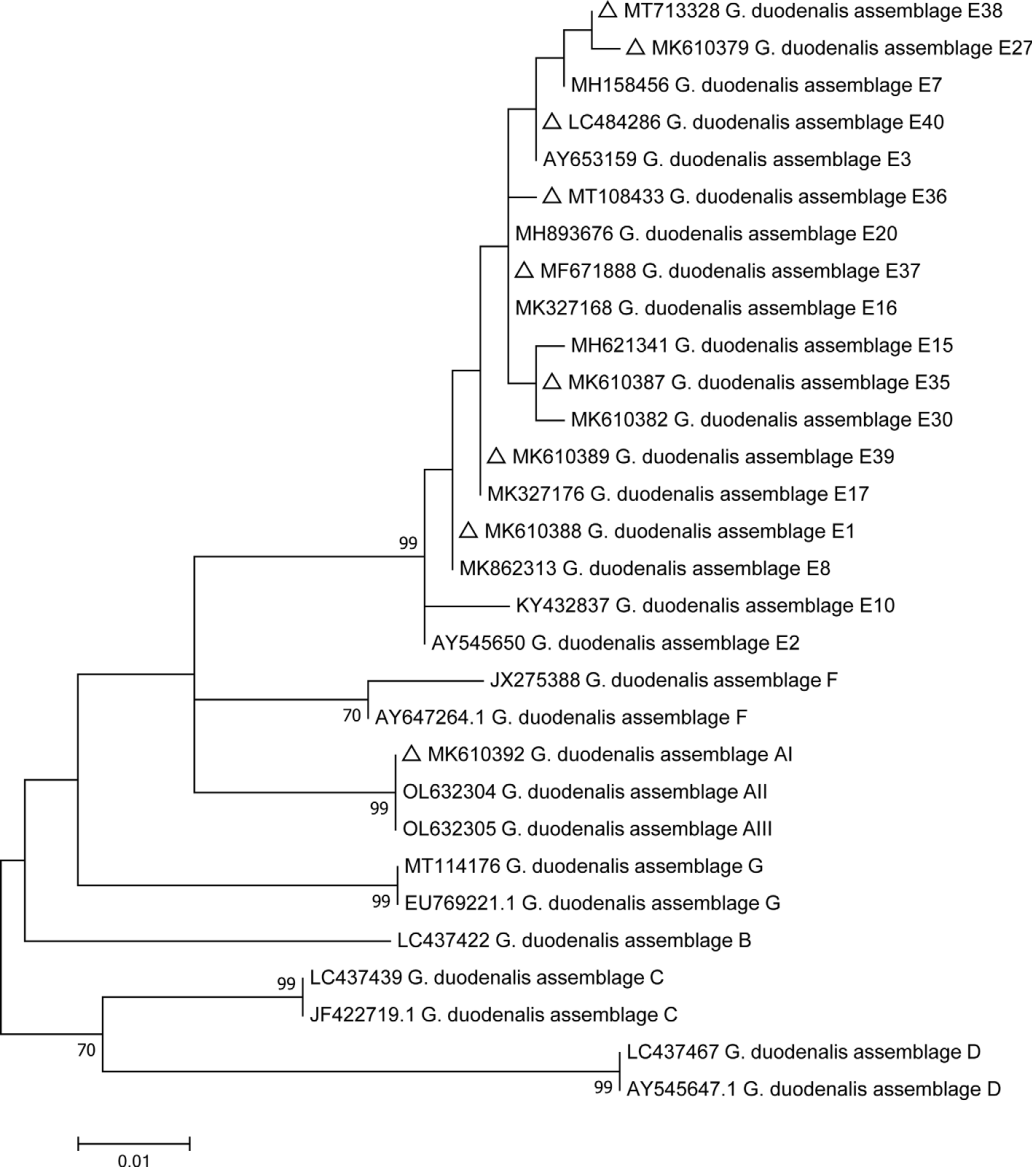
Phylogenetic relationships of beta-giardin (*bg*) nucleotide sequences of *G. duodenalis* assemblages (A–G) and assemblage E subtypes, using the maximum composite likelihood model. Percent bootstrap values greater than 50% from 1000 replicates are shown next to the branches. The hollow triangles represent published isolates in this study.

**Figure 3:**
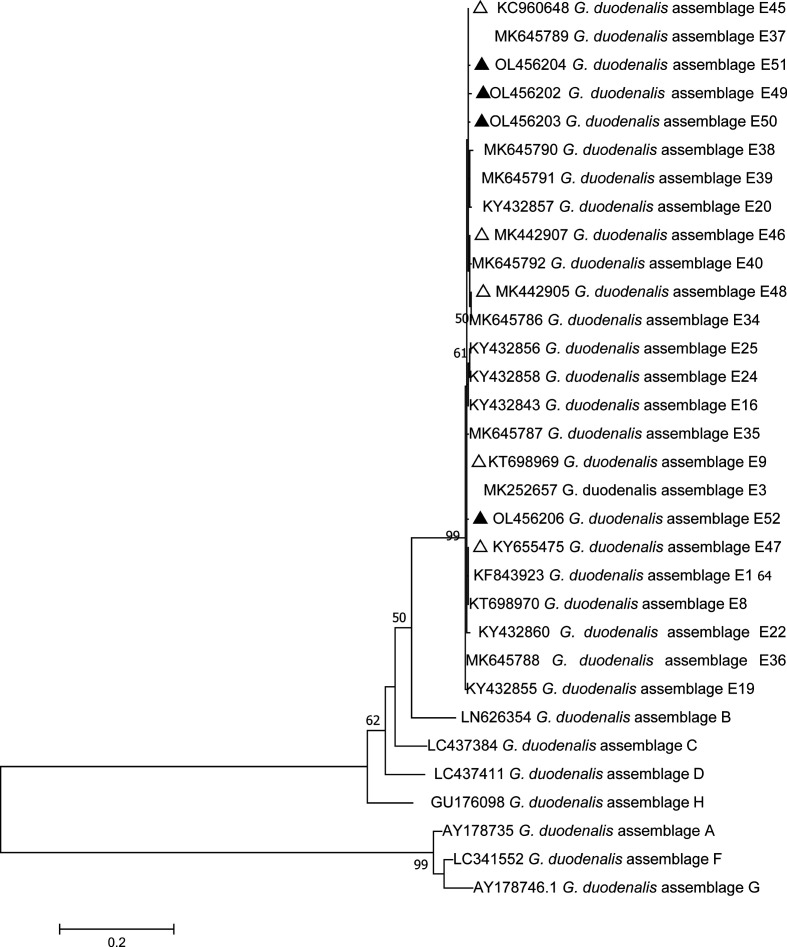
Phylogenetic relationships of glutamate dehydrogenase (*gdh*) nucleotide sequences of *G. duodenalis* assemblages (A–H) and assemblage E subtypes, using the maximum composite likelihood model. Percent bootstrap values greater than 50% from 1000 replicates are shown next to the branches. The black triangles and hollow triangles represent published and novel isolates in this study.

**Figure 4:**
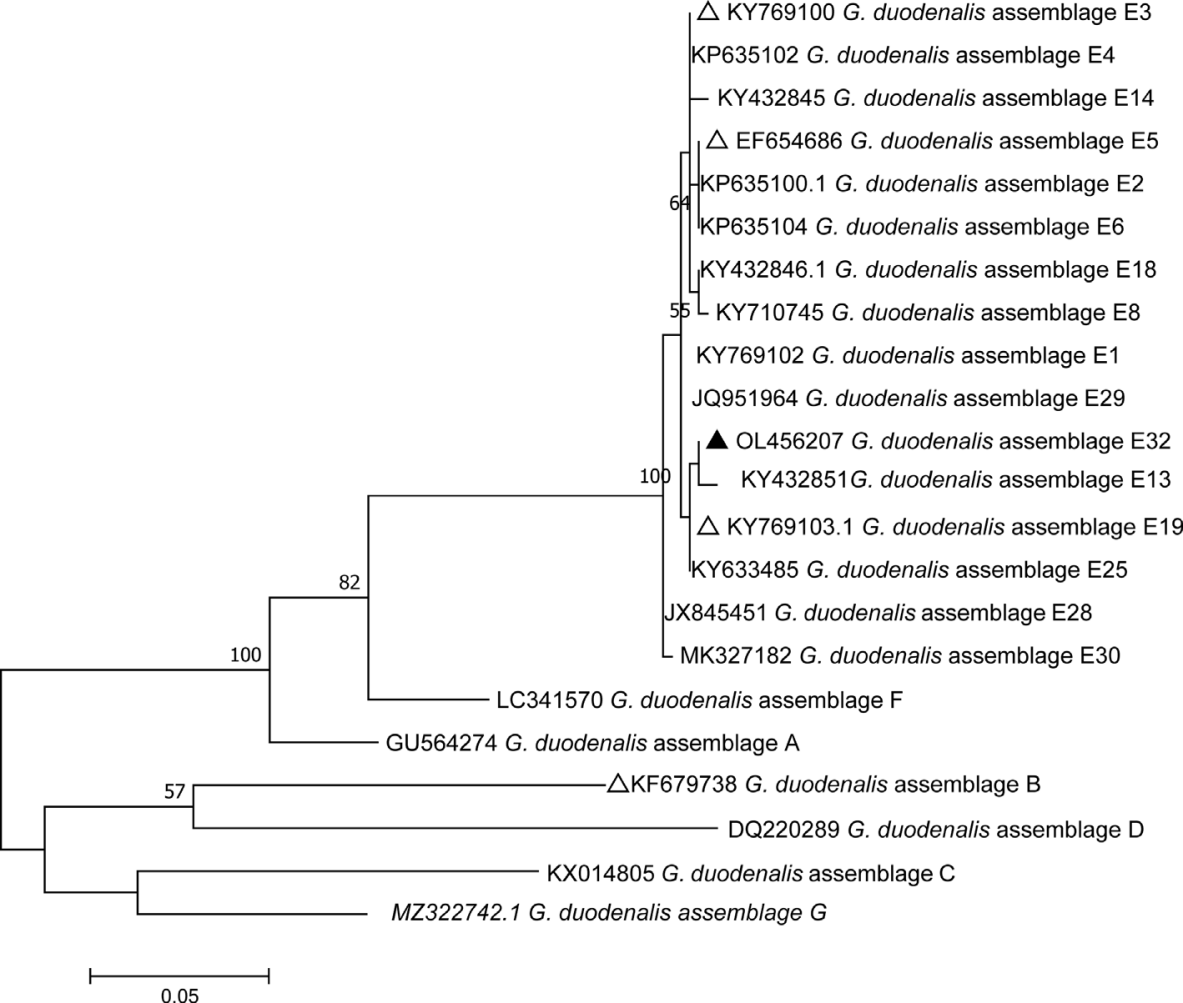
Phylogenetic relationships of triose phosphate isomerase (*tpi*) nucleotide sequences of *G. duodenalis* assemblages (A–G) and assemblage E subtypes, using the maximum composite likelihood model. Percent bootstrap values greater than 50% from 1000 replicates are shown next to the branches. The black triangles and hollow triangles represent published and novel isolates in this study.

Based on the *G. duodenalis bg*-sequences, *gdh*-sequences and *tpi*-sequences, three phylogenetic trees were constructed to evaluate the genetic relationships of the *G. duodenalis* isolates. The results showed that *G. duodenalis* isolates from this study were clustered within the *G. duodenalis* assemblage E, and high genetic diversity was observed in the assemblage E subtypes (Figs. 2–4).

## Discussion

This study presented *G. duodenalis* distribution in sheep, goats and beef cattle in Southwest Inner Mongolia. *Giardia duodenalis* in this study were detected by *bg* locus, and the total infection rate was 4.0%. In previous reports using the same method, there was a higher *G. duodenalis* infection rate in Tan sheep in northwestern China (10.95%) [22], cattle in Turkey (30.2%) [21], beef cattle in Scotland (10.1%) [3], Tibetan sheep (13.1%) and yaks (10.4%) in Qinghai province, China [14]. However, there was a similar infection rate in healthy adult domestic ruminants in central Iran (5.2%) [15], and sheep in Inner Mongolia, China (4.3%) [34], which were detected by the *tpi* locus. Based on the *SSU rRNA* gene, *G. duodenalis* was detected in livestock in the United Kingdom (34.3%) and sheep in Inner Mongolia, China (64.1%) [6, 18].

The *SSU rRNA*, *bg* and *tpi* loci have frequently been used to detect *G. duodenalis*. In this study, *G. duodenalis* in fecal samples was detected by nested-PCR of the *bg* locus, and only 29.3% and 10.3% *bg*-positive samples were amplified based on the *gdh* and *tpi* loci, which were similar to previous studies [3, 14, 21, 22]. The difference between the *G. duodenalis* infection rate in this study and that in other studies which used the *bg* locus may be partially attributed to the state of feces, age group, sample size, detection methods and climate.

All samples in this study were collected from non-diarrhea livestock in the age groups of seven months and older. The *G. duodenalis* infection rate was significantly different between different age groups of beef cattle (*p* < 0.001). Previous studies showed a higher prevalence in sheep, goats and cattle before weaning, and *G. duodenalis* infection is inversely associated with animal age [8, 17, 35]. The *G. duodenalis* infection rates in winter were significantly higher than in summer and autumn (*p* < 0.01), and the same phenomenon was reported in dairy calves in Norway and pigs in Denmark [13, 23]; however, the season was not significantly associated with giardiasis infection of yaks in Qinghai, China [26].

*Giardia duodenalis* assemblages A, B and E were identified, and *G. duodenalis* assemblage E was the dominant assemblage found in this study, which is consistent with previous reports [6, 7, 25]. *Giardia duodenalis* assemblages A and E were identified as the two most common assemblages in sheep, goats and cattle, with assemblage B reported occasionally [11, 25, 35, 36]. A few studies have reported assemblage C and assemblage D in livestock, but it is unknown whether this was an actual infection or mechanical transmission [15, 18, 20, 31]. The *G. duodenalis* assemblages in this study were also reported in humans, companion animals and wildlife [24], and more research is needed to verify the potential impact on public health safety.

High genetic diversity was observed in the assemblage E subtypes. At the *bg* locus, eight published assemblage E subtypes were found in sheep, goats and beef cattle, and the *bg*-positive samples were analyzed by the multilocus genotyping tool with high resolution (*gdh* and *tpi*) to further reveal the genetic variations in *G. duodenalis*. A total of four and one novel assemblage E subtypes were found at the *gdh* and *tpi* loci, respectively and the analysis yielded four novel MLGs of assemblage E. A high degree of genetic diversity in *G. duodenalis* assemblage E has been reported in livestock, which was probably a cause of the high occurrence rate of *G. duodenalis* in Tibetan sheep and yaks [14, 32]. In this study, the same *G. duodenalis* assemblage E subtypes (E1, E35 at the *bg* locus and E45 at the *gdh* locus) were found in sheep, goats and beef cattle simultaneously, which may indicate a potential occurrence of cross-species transmission. Cross-species transmission of *G. duodenalis* assemblage E subtypes was also found in Tibetan sheep and yaks [14], black-boned sheep and black-boned goats [7].

## Conclusion

The results of this study show that *G. duodenalis* is a common parasite in sheep, goats and beef cattle in Inner Mongolia, and the infection rate is related to the season, and age of beef cattle. Based on molecular analysis, three *G. duodenalis* assemblages (A, B and E) were found and assemblage E was predominant. Novel subtypes found in this study show further genetic diversity of *G. duodenalis* assemblage E. This study provides baseline data for preventing and controlling *G. duodenalis* infection in livestock.
